# Silicodata: An Annotated Benchmark CXR Dataset for Silicosis Detection

**DOI:** 10.1038/s41597-025-05595-4

**Published:** 2025-09-26

**Authors:** Yasmeena Akhter, Rishabh Ranjan, Mayank Vatsa, Richa Singh, Santanu Chaudhury, Anjali Agrawal, Shruti Aggarwal, Arjun Kalyanpur, Anurita Menon

**Affiliations:** 1 Department of Computer Science and Engineering, Indian Institute of Technology Jodhpur, Jodhpur, 342030 India; 2 Department of Electrical Engineering, Indian Institute of Technology Delhi, 110016 New Delhi, India; 3 Teleradiology Solutions, Bangalore, 560048 India; 4 Image Core Lab, Bangalore, 560048 India

**Keywords:** Nutrition, Electrical and electronic engineering, Whole body imaging, Public health

## Abstract

This research introduces a unique dataset targeting Silicosis, a significant global occupational lung disease, and a member of the Pneumoconiosis family. Addressing the challenges in healthcare data collection and the need for expert annotation, this dataset aims to aid AI algorithms in medical applications. The comprehensive dataset includes not only Silicosis cases but also related conditions, such as tuberculosis and silicotuberculosis, alongside healthy lung images, addressing the diagnostic complexity due to symptom overlap. As the first public dataset of its kind, it offers detailed annotations for lung and disease region segmentation, as well as disease prediction, provided by multiple radiologists. Baseline experiments and findings demonstrate that current AI models have limited predictive accuracy for these disease classes, emphasizing the critical need for dedicated research. It is our assertion that the proposed Silicodata can be a key dataset in designing automated Silicosis detection tools and addressing challenges associated with small sample sizes in medical AI research.

## Background & Summary

The rise of industrialization and the growing demand for raw materials and heavy machinery have led to the creation of various hazardous work environments. These industrial sites release a wide range of harmful substances, including toxic gases such as sulfur dioxide and nitrous dioxide, solid pollutants such as arsenic, lead, and chromium, and hazardous liquids such as mercury. This proliferation of dangerous materials has led to an increase in occupational lung diseases, particularly pneumoconiosis, a group of lung conditions caused by inhaling dust at work. Silicosis, a specific type of pneumoconiosis, results from prolonged exposure to respirable crystalline silica dust. Silica, a natural mineral found in rocks, sand, and soil, is widely used in industries like construction, mining, and foundry work. Workers exposed to silica dust risk developing silicosis, an irreversible and sometimes fatal lung disease characterized by lung scarring, impaired breathing, coughing, wheezing, and shortness of breath.

Silicosis is especially prevalent among workers in certain African and Asian countries, including India. The disease increases the likelihood of developing pulmonary tuberculosis^1–4^ and post-tuberculosis fibrosis. Patients with silicosis are significantly more likely to develop tuberculosis than those without. In some cases, silicosis co-occurs with tuberculosis, leading to silicotuberculosis^5,6^. Globally, millions of workers, especially in small-scale industries, are at risk of silicosis^7,8^. Despite efforts to eradicate Silicosis by 2030, as targeted by the WHO and ILO, the rising number of industrial sites and increased worker involvement suggest that this goal may be difficult to achieve. Preventive measures in workplaces, particularly in low-to-middle-income countries, often suffer from poor implementation and limited awareness.

**Fig. 1:**
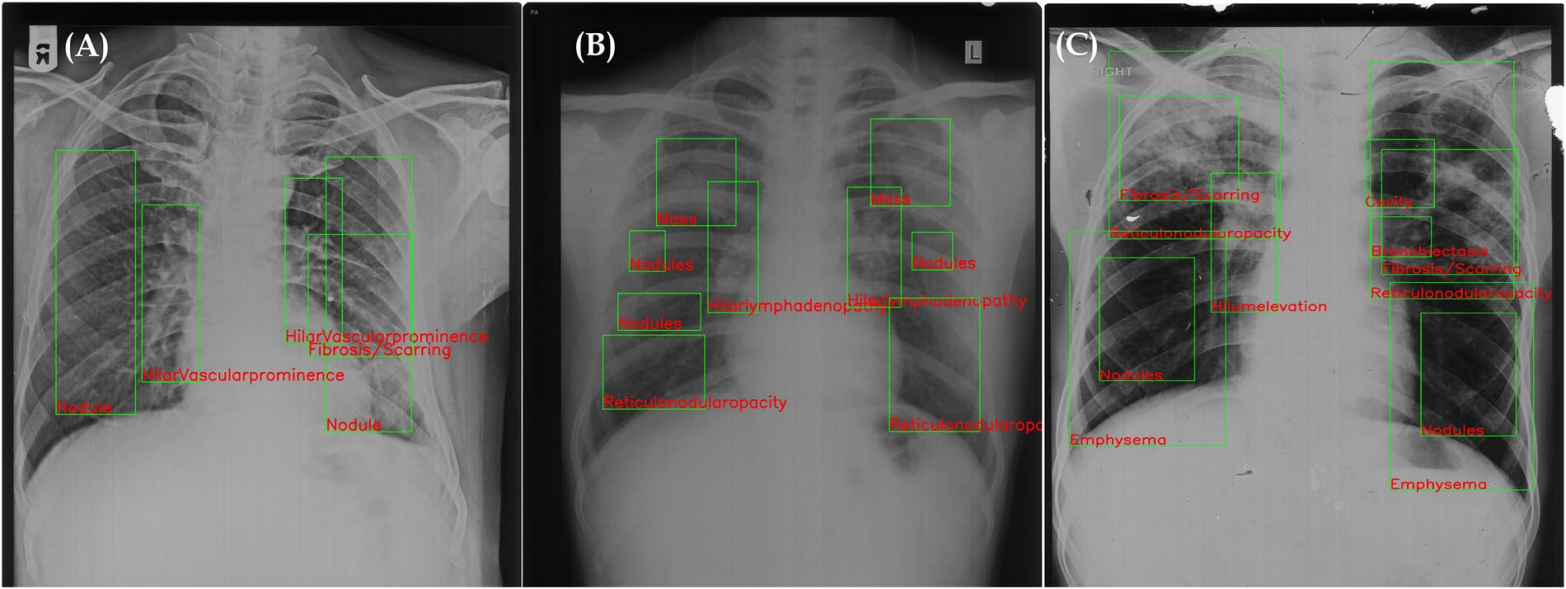
Sample chest X-rays of individuals with (**A**) Silicosis, (**B**) Silicotuberculosis, and (**C**) Tuberculosis, highlighting different manifestations present for three disorders.

The integration of AI in medical image analysis, particularly in computer-aided diagnosis (CAD), has become increasingly prominent, especially in under-resourced rural areas. However, research in pneumoconiosis detection, especially AI-driven Silicosis identification from chest X-rays, faces significant limitations^9–16^. These include a scarcity of publicly accessible datasets and an over-reliance on limited in-house sample size datasets. This situation highlights a critical research gap in AI-based Silicosis diagnosis, characterized by several challenges:

- **Small Sample Size:** Unlike more prevalent conditions such as Tuberculosis and Pneumonia, there is a scarcity of labelled silicosis images for training machine learning models. Pre-trained models heavily rely on large-scale labelled data for effective transfer learning. Without sufficient silicosis-specific data, these models cannot be fine-tuned adequately, leading to suboptimal performance. Most studies rely on a small set of samples, limiting the robustness and generalizability of AI algorithms. Moreover, these studies often fail to ensure interoperability among diverse sample sets.
- **Diagnostic Overlap and Co-morbidity:** Silicosis symptoms often mimic those of other lung diseases like tuberculosis (TB), leading to potential misdiagnoses. Moreover, Silicosis patients frequently suffer from concurrent lung disorders, with a common co-occurrence of TB, resulting in conditions like Silicotuberculosis.
- **Subtle and Heterogeneous Radiographic Features**: Silicosis manifests with subtle radiographic features such as pulmonary opacities and nodules as shown in Fig. 1(A), which can be small and difficult to detect. These features exhibit significant heterogeneity due to variations in:
  - **Acquisition Modalities**: Differences in imaging equipment and settings can affect image quality and appearance.
  - **Imaging Protocols**: Variations in protocols between institutions lead to inconsistencies in image characteristics.
  - **Patient Demographics**: Factors like age, body habitus, and co-existing conditions can alter radiographic presentations.
- **Lack of Detailed Annotations and Radiology Reports:** Detailed annotations for each finding are crucial for guiding AI models, but creating these is labor-intensive and requires medical expertise. Current research lacks such annotations for automated AI-based Silicosis detection. Additionally, associated radiology reports are often unavailable.
- **Out-of-Domain Challenges and Variability in Imaging Techniques:** In many rural areas, conventional screen-film radiography (SFR) remains in use due to limited access to advanced healthcare. This results in lower-quality scans compared to digital radiography, posing a challenge for accurate AI analysis. Variations in image resolution, contrast, and noise levels can create out-of-domain challenges for pre-trained models, which are not equipped to handle these discrepancies without appropriate adaptation.
- **Loss of Critical Features Due to Image Downscaling**: Pre-trained models often require input images to be resized to smaller dimensions (e.g., from 3K × 3K pixels to 224 × 224 pixels) to fit the network architecture. This downscaling can lead to the loss of critical radiomic features, such as small nodules or opacities, which are essential for accurate silicosis detection. The reduction in image resolution diminishes the model’s ability to recognize these subtle patterns.
- **Issues in Creating Large Dataset for Silicosis:** Compiling a substantial dataset with accurate annotations is a formidable and time-intensive endeavor. As an illustration, the ChestX-ray8^17^ dataset required approximately 23 years to assemble. Adopting a similar methodology for Silicodata, limited participation and eligibility for radiological studies, and restricted data-sharing permissions are two primary issues. The process of sharing sensitive medical data necessitates adherence to stringent protocols. Typically, radiographs feature identifiable information, such as the patient’s name and the hospital, on one of the margins. Moreover, hospital data often includes X-ray images of various body parts, necessitating significant time and effort to filter and organize images according to the specific organ targeted for study. This issue was similarly encountered in the collection process for Silicodata, necessitating manual exclusion of non-chest X-ray images to ensure dataset relevance and integrity.

To address the challenges in AI-based diagnostic solutions for Silicosis, we introduce the *Silicodata* benchmark chest X-ray dataset, designed to automate Silicosis detection. The key contributions include the creation of *Silicodata*, a novel dataset featuring global labels for Silicosis, silicotuberculosis, TB, and normal cases. Additionally, a subset of this dataset includes lung field masks as ground truth and detailed annotations by expert radiologists, providing local labels for specific lung findings. This can not only enhance disease region detection accuracy but also add explainability to the results. The subset further comprises radiology reports, facilitating the development of automated report-generation algorithms. We also outline detailed protocols for segmentation and classification, accompanied by baseline results for these tasks on Silicodata. The public release of this dataset and its annotations aims to encourage researchers to work on this critical and socially relevant problem and aid in a fast and reliable early diagnosis of Silicosis. The major contributions to solving the challenging AI-based detection of Silicosis are summarized below:

- **Meticulous Collection and Annotation**: We have meticulously collected and annotated a comprehensive and first-of-kind set of radiographs specifically related to Silicosis, Silicotuberculosis and Tuberculosis. Our dataset captures the distinct features associated with the condition, ensuring the subtle differences between Silicosis, Silicotuberculosis and Tuberculosis and enables the development of models that are more sensitive and accurate in detecting and differentiating silicosis with other related diseases.
- **Demonstrating the Importance of Domain-Specific Adaptation**: Our findings highlight that while pre-trained models serve as a valuable foundation for transfer learning, their performance is highly dependent on dataset-specific characteristics. Domain-specific adaptation is crucial for models to perform effectively in specialized tasks like silicosis detection.
- **Detailed Radiology Reports**: Report generation for Silicosis analysis can impart a major impact to recognize that automated report generation could greatly enhance clinical workflows and patient care. The current version of *Silicodata* paves a path for report generation for Silicosis.

We believe that our work bridges the gap by offering a valuable resource for developing more effective machine-learning models tailored to silicosis detection. By facilitating domain-specific adaptation, we aim to enhance diagnostic accuracy and contribute to better clinical outcomes.

## Methods

This research introduces the first public dataset aimed at facilitating Silicosis detection. It comprises 3, 044 frontal chest X-rays (CXR) collected retrospectively across three years from stone workers at primary health centers in Rajasthan, India. The dataset encompasses cases of Silicosis, Silicotuberculosis (STB), Tuberculosis (TB), and normal conditions. The distribution of samples pertaining to the four classes in the proposed *Silicodata* is summarized in Table 1. The average size of each sample is around 3567 × 2898, provided in grayscale JPEG format. The dataset is structured into two distinct subsets. Set A comprises images that are tagged with disease labels only; these are known as global labels. Set B includes images that not only have disease labels but also have lung segmentation maps and detailed annotations (referred to as local labels). The samples for Silicosis, STB and TB, along with the corresponding findings, are highlighted in Fig. 1.

**Table 1:** Distribution of the proposed Silicodata.

Partition	Silicosis	STB	Tuberculosis	Normal	Total
Train Set	738	529	525	337	2129
Test Set	319	227	225	144	915
Total	1057	752	750	481	3044

### Ethical statement

The Silicodata consists of human data in the form of CXRs from patients. The data collection protocol has been approved by the Institutional Review Board (IRB) of IIT Jodhpur, India (Ref No. IEC/IITJ/2022-23/03). The required permissions have been obtained from the authorities involving medical officers in hospitals and public health centers where data is collected. Consent from the patient is waived off with proper permission from the Institute Ethics Board and hospital administration, as the collected data is stored and shared anonymously for data annotation and is completely deidentified for future release. Hence, no personal information about the subject (patient) or the name and address of the data collection centers is collected or released.

### Image Selection and Preprocessing

The proposed dataset has been meticulously assembled from samples collected retrospectively across a three-year span from various hospitals. During this period, physicians recommended chest radiography for patients exhibiting symptoms such as chronic cough, shortness of breath, fever, breathing difficulty, and cyanosis. In the process of curating the data, we prioritized image quality, excluding any samples deemed noisy or of poor quality after thorough review and input from expert radiologists. Note that image quality is paramount in the tasks of segmentation, disease detection, and classification, especially since chest X-rays are grayscale images where any form of preprocessing can significantly impact the saliency attributes of the samples. Furthermore, the orientation of the image, due to rotation during the capture process, is crucial for emphasizing specific anatomical structures and can greatly influence the interpretability of the radiographs. These factors, rotation and image quality, are critical challenges we addressed in the data collection phase, discarding any samples that were overexposed or unreadable. In the final dataset, the rotation ranges from none to mild, and we have resized the images to 224 × 224 pixels for both segmentation and classification purposes to minimize computational demands. Initially collected in DICOM format, the images have been converted to JPEG to maintain consistency with existing chest X-ray datasets. Silicodata features frontal CXRs from both digital and conventional scans, with the latter being digitized. This approach introduces a diversity in the dataset, especially beneficial for regions where digital X-ray facilities are scarce.

### Data De-Identification

To safeguard patient confidentiality, all identifiable details, including names, addresses, hospital locations, dates of acquisition, and physician identities, have been meticulously stripped from the CXRs. The original DICOM images were transformed into JPEG format via a Python script, during which process all sensitive patient information was excised. The dataset retains only images being renamed according to a new, anonymized naming scheme. Furthermore, for images originating from conventional screen-film radiography (SFR), we applied cropping techniques to ensure the removal of any information that could potentially identify patients. This approach ensures the privacy of the patients while maintaining the utility of the dataset for research purposes.

### Data Annotation Procedure

**Fig. 2:**
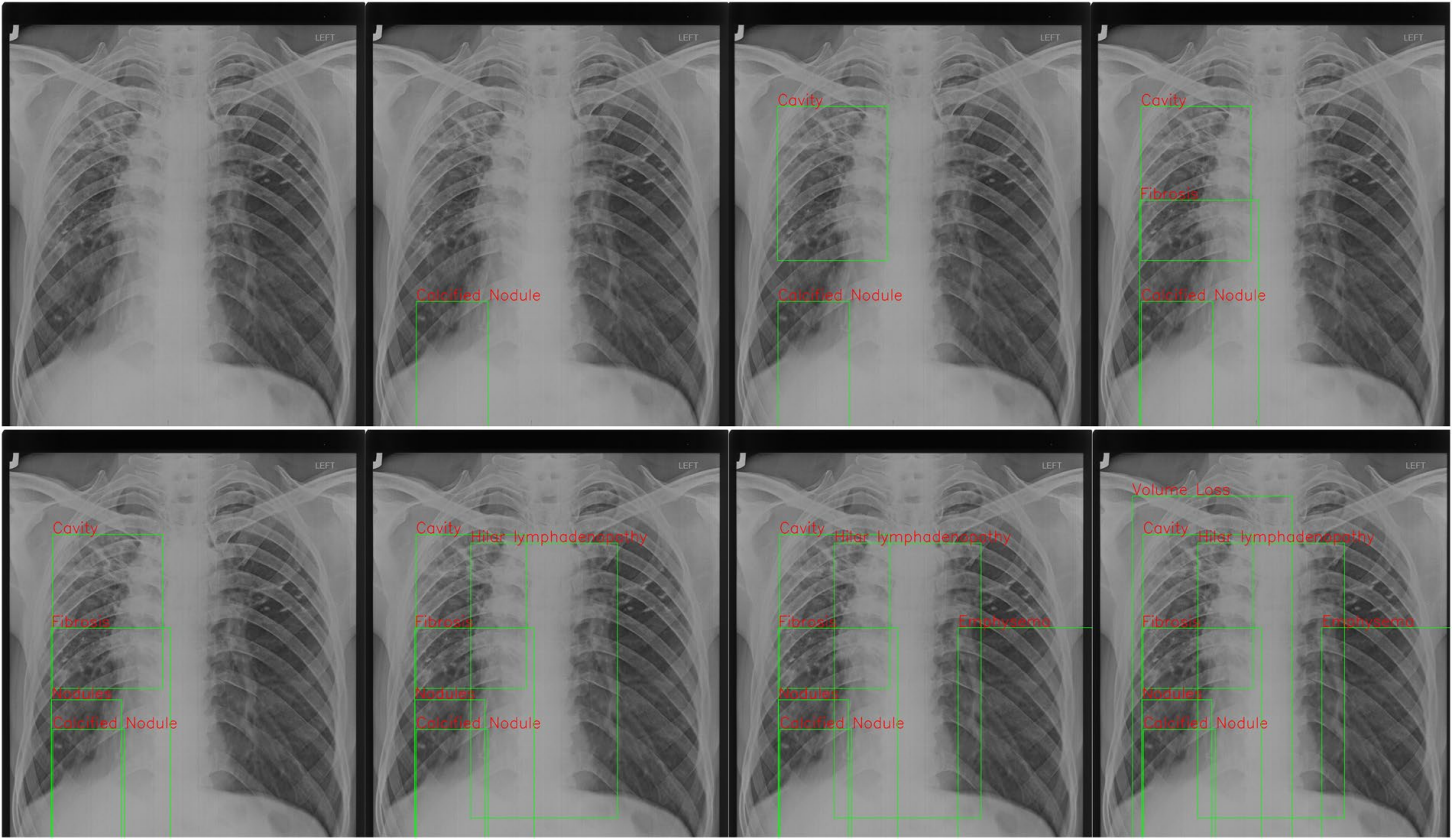
Showcasing the annotations for different findings for a given chest X-ray image. Each finding is shown as a bounding box and effectively highlights the annotation methodology and provides valuable insight into the range of findings that can possibly exist in the Silicosis and related disorder-affected Chest X-rays. The Figure showcases the progression of each finding to be clearly visualized without interference from other annotations and effectively demonstrates how each annotation appears on the image, providing a clearer understanding of the localization of different findings.(Best viewed in colour).

**Fig. 3:**
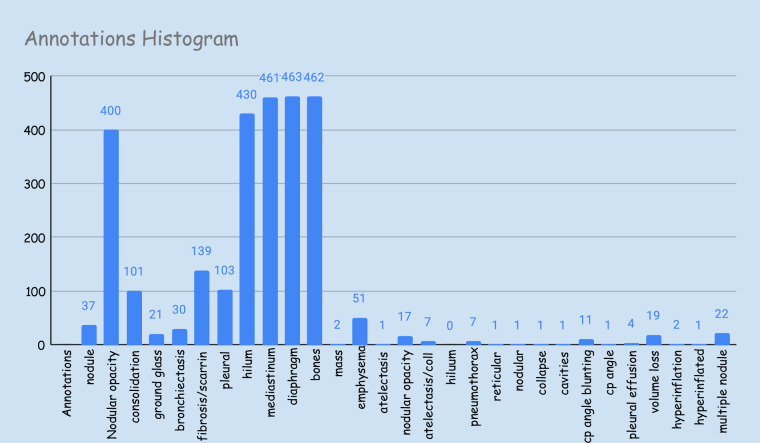
Histogram of different annotated findings present with Set B radiology reports of Silicodata. It can be found that the frequency of the findings is variable across the samples and becomes challenging for local label-based classification due to class imbalance, particularly in multi-label, multi-class settings.

For the annotation of this dataset, we partnered with Tele-radiology Solutions in Bangalore, India. A team of five board-certified radiologists, using the LabelImg toolbox, meticulously performed the annotation process, ensuring the elimination of any biases in decision-making. To ensure consistency and minimize variability in annotations among the radiologists, the team was divided, with each member responsible for a portion of the images. A significant measure to further ensure uniformity was the organization of a discussion and training session led by the two senior radiologists for the remaining three, followed by a review process that covered about 20% of the cases. The annotated data is provided in XML format for detailed chest findings, with an additional conversion into .docx format for enhanced accessibility, as illustrated in Fig. 2. The dataset documents a range of findings, including, but not limited to, nodules, consolidation, ground glass opacity, and pleural effusion, among others. Figure 3 shows the distribution of the local labels in set B. The categories of global labels are Silicosis, STB, TB, and normal, presenting a comprehensive framework for addressing this complex multi-label, multi-class research challenge. Annotation in this context is notably intricate, even for experts, due to the variable quality of digital imaging, which, as reported in the literature, generally surpasses traditional screen-film radiography^18^ in clarity and detail. The *Silicodata* faced challenges related to the quality of some images, which were not digital and exhibited limitations such as chemical artifacts or damage due to poor storage, affecting the evaluation of these images. These limitations and their impact on the assessment have been duly noted in the corresponding reports, highlighting the rigorous approach to dataset annotation. Annotations done for both local and global labelling encourage solutions for the challenging multi-label, multi-class research problem.

**Fig. 4:**
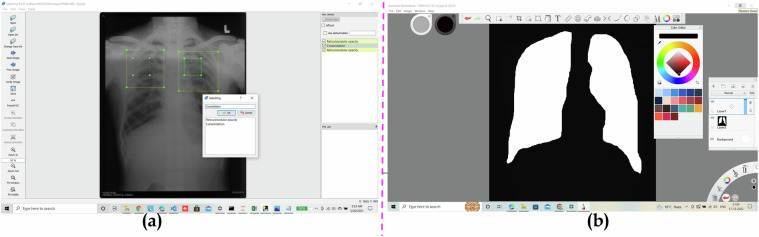
Showcasing the Graphical User Interface of Labellmg (**a**) and Sketchbook software (**b**). Both (**a**) and (**b**) demonstrate how the bounding-based annotation for locating the findings and drawing the segmentation masks around the lung fields was generated, respectively.

**Fig. 5:**
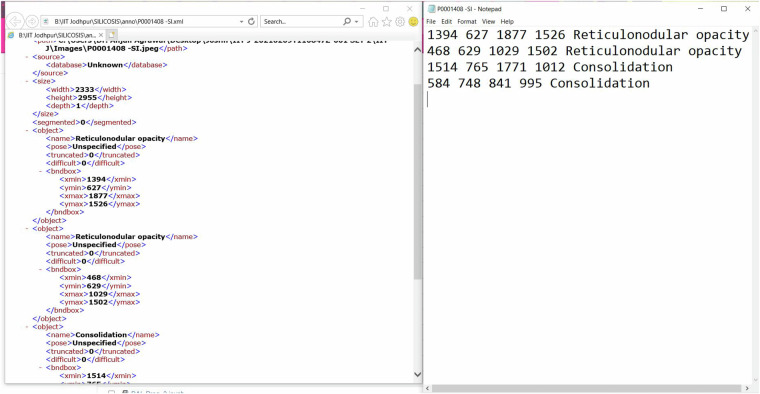
Illustrates an example of the annotations on **left** in XML format. A bounding box is drawn for each finding in the chest X-ray image, and the four coordinates for two corner points are reported in the XML file. In the given XML file, it can be seen that ‘name’ specifies a particular finding and (*x*_*min*_, *y*_*min*_, *x*_*max*_, *y*_*max*_) as coordinates. On **right**, its simpler version is extracted as a text file with four coordinates followed by the label of the bounding box.

**Fig. 6:**
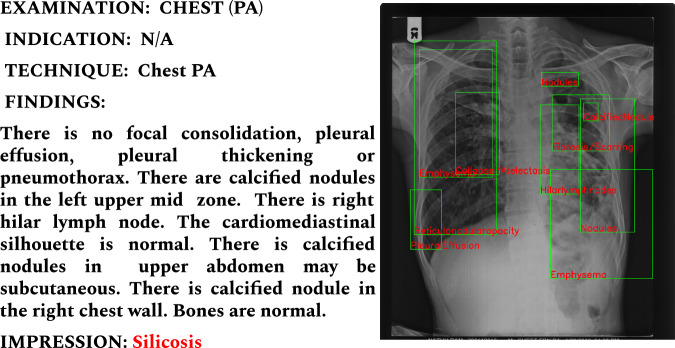
Showcases the samples of annotated images for different findings. On the right, we have a radiology report highlighting different findings along with the impression (Silicosis, STB, TB, or Normal). The report includes both the local labels, such as nodule, consolidation, emphysema, and many more, along with the global label, Silicosis.

### Annotation Tool

To generate the segmentation masks and findings for set B, we used *Sketchbook* and *Labellmg* software, respectively. To perform the annotations for different findings in a given CXR, we used the Labellmg tool. Labellmg is a graphical image annotation tool. It is written in Python and uses Qt for its graphical user interface. Annotations are saved as XML files in PASCAL VOC format. Besides, it also supports YOLO and CreateML formats. Figure 4(a) showcases the GUI of the Labellmg tool. Figure 5 shows a sample of annotations in XML format and its corresponding text formats. To generate the ground-truth mask of the lungs for set B, domain experts manually generated the mask using the Sketchbook software. We opted for a fully manual approach to ensure the precision of the annotations. This meticulous process was crucial to accurately delineate the lung boundaries, especially given the subtle and complex patterns associated with silicosis. Manual annotation allows radiologists to incorporate their expert knowledge and interpret nuanced imaging features that automated segmentation algorithms might overlook or misinterpret. Figure 4(b) showcases the GUI of the Sketchbook software. Both **(a)** and **(b)** demonstrate how the bounding-based annotation for locating the findings and drawing the segmentation masks around the lung fields was generated, respectively.

## Data Records

The proposed Silicodata is hosted publicly on the Harvard Dataverse portal^19^. The dataset consists of 3044 samples and is organized into two folder sets - set A and set B:

- **Set A:** It contains the images with only disease labels referred to as global labels
- **Set B:** It contains images with lung segmentation maps, annotations, and disease labels, referred to as local labels

Set A comprises all the 3044 samples in the dataset, assigned with the global labels for four class classification tasks. On the other hand, Set B, a subset of the dataset, comprises 445 samples, assigned with local labels using detailed annotations. The cohort consists of 2334 male subjects aged 16 to 60 and 435 female subjects aged 20 to 55. The stone-working industry, particularly in sectors such as mining, has historically been male-dominated in many regions, including the area from which our data is sourced. Due to these occupational factors, silicosis is predominantly observed in males and largely explains the skewed gender distribution in our dataset. This disparity accurately reflects the real-world demographics of silicosis among stone workers. Depending on the number of visits of each subject, the dataset contains a different number of images per subject.

Set A consists of samples with four classes assigned with a global label for Silicosis, STB, TB, and Normal. Set B contains a set of Chest X-ray samples containing the detailed annotation for the findings referred to as local labels. The local labels include Atelectasis, Cardiomegaly, Consolidation, Edema, Enlarged cardiomediastinum, Fracture, Lung lesion, Lung opacity, No findings, Pleural effusion, Pleural other, Pneumonia, Pneumothorax, Nodules, Nodular opacity, Cavity, Bronchiectasis, Hilum, CP angle, and Volume loss. Moreover, Set B contains the segmentation ground truth and detailed reports as well. The reports contain the view information, details of the overall chest X-ray for local labels, and the impression keyword represents the global label.

For set B, we also include a CSV file containing the filename and the findings regarding the local and global labels. The annotations for Set B and image quality corresponding to CXR images are provided in ‘Silicodata_SetB_labels.csv’. Each row in the CSV file specifies the image filename, its class (Silicosis/STB/TB/normal), the quality of the CXR image, and all the findings in the CXR. For set B, we include the lung field ground-truth mask in a separate folder titled ‘set_B_masks’. For set A, we include two folders with the naming convention as train_images and test_images. These folders further contain four subfolders for each of the classes, with a naming convention as ‘folder_class’ (class: Silicosis/STB/TB/normal). More details follow below:

- There is a primary folder named ‘Silicodata_dataset’, which contains two subfolders, set A and set B.
- The subfolder ‘set_A_folder’ contains the images of set A. It further contains two subfolders as train_images and test_images, and each contains four subfolders - ‘folder_Silicosis’, folder_STB’, ‘folder_TB’, and ‘folder_normal’.
- The subfolder ‘set_B_folder’ contains subfolders for images, annotations, and a CSV file. These subfolders are titled ‘set_images’, ‘set_B_Annotation_XML’, ‘set_B_Annotation_Text’, and ‘set_B_masks’. set_images contains images of set B. set_B_Annotation_XML contains the bounding box information for each image in set B in XML format. set_B_Annotation_Text contains the same annotations for each finding in text format. set_B_masks folder contains all the ground-truth masks for lung fields. set_B_folder also contains a CSV file named ‘Silicodata_SetB_labels.csv’.

By making the Silicodata dataset publicly accessible, we aim to inspire new line of research that addresses the current challenges in early Silicosis detection. Additionally, it opens up opportunities for further exploration within the dataset, including computer vision tasks like explainability, fairness, modelling with low-resolution data, federated learning, and more.

### Radiology Reports

For subset B, each data sample is accompanied not only by annotations but also by anonymized radiological reports. The anonymization process involved assigning a unique identifier to each case prior to evaluation by radiologists. These reports, provided in Word document format, detail the observations found within the radiographs, including specific locations. They are structured using a template that catalogs potential chest abnormalities, such as nodules, consolidation, pleural effusion, calcified nodes, and cavitation. Radiologists then indicated the presence or absence of each listed finding, culminating in a diagnostic conclusion that facilitated the categorization of each case. Importantly, these reports exclude any personal or hospital-related information, maintaining patient confidentiality. The radiologists were not given any identifying information, ensuring the reports’ complete anonymity.

An example from the Silicodata set (in Fig. 6), showcases a report alongside its corresponding annotated image, highlighting findings within various sections of the thoracic cavity such as the diaphragm, hilum, mediastinum, heart, and bones. Our dataset provides the scope of report generation for silicosis analysis, thus enabling automatic report generation, which could greatly enhance clinical workflows and patient care. However, to date, no studies have specifically focused on the generation of radiological reports for diagnosing Silicosis through this approach. This dataset can be an important asset for generating comprehensive reports for chest X-rays that not only cover Silicosis but also other thoracic conditions, similar to the capabilities of existing Open-i datasets^20,21^.

### Dataset Documentation and Availability

As mentioned earlier, the proposed dataset is made available at Harvard Dataverse^19^. The annotations and associated finding labels for set B images are provided in the XML file. We also provide a simpler version of the annotations as text files containing the coordinates of the bounding box and its corresponding finding (Local Label). Each image has one XML and one corresponding text file. The XML and text files are saved with image filenames and contain rectangular bounding box coordinates and related radiological findings. Each row corresponds to one bounding box annotation, and one CXR image may have multiple such annotations. After signing the license agreement, the files can be downloaded directly from the above link.

## Technical Validation

Using the proposed Silicodata dataset, we have established benchmarks for both segmentation and classification tasks, tailored to two distinct scenarios. Our approach to classification encompasses both binary and multi-class models aimed at detecting Silicosis in chest X-ray images. The success of automatic disease identification hinges on accurately locating the distinctive features within the images that are indicative of the disease. Consequently, segmenting the lungs from the CXR images is a critical step, allowing algorithms to concentrate on the image areas most relevant to disease detection. To this end, we have conducted baseline experiments for both segmentation and classification tasks using the experimental protocol shown in Table 2.

**Table 2:** Illustrates the protocol for training and testing for segmentation and classification experiments.

S.No	Protocol	Classes	Training + Validation	Test
1	Lung Segmentation	Lung Region	704	445
		Other Region		
2	4 class classification	Normal	337	144
		Tuberculosis	525	227
		Silicosis	738	319
		SilicoTuberculosis	525	225

For the segmentation task, the training images are taken from Shenzen and Montgomery^22^, and models are tested on set B of the proposed Silicodata. The classification task is done on the combined samples of set A and set B.

### Segmentation Task

Diagnosing Silicosis using chest X-rays (CXR) is challenging due to the scarcity of publicly available datasets, which represents a small sample size challenge. This scarcity often leads to overfitting and suboptimal generalization when training deep learning models for such specific tasks. To mitigate these challenges, domain adaptation and transfer learning have become prevalent strategies, particularly in the medical field. In our study, we adopted transfer learning for both segmentation and disease classification tasks to address the small sample size challenge inherent in silicosis detection from CXRs. We leveraged the Shenzen and Montgomery datasets^22^ as the source data and Silicodata as the target data for the segmentation tasks.

Deep learning-based models present state-of-the-art results in segmentation and classification. In line with this, we implemented two widely recognized deep learning models, SegNet^23^, and UNet^24^, for segmenting samples in Set B of Silicodata, which includes ground-truth segmentation masks. For both the models, ResNet50^25^ pre-trained on ImageNet^26^ is used as encoder layers, focusing on semantic segmentation of lung fields. The training utilized samples from the Montgomery County and Shenzhen datasets^22^, both of which offer publicly available lung masks. We applied various data augmentation techniques, such as horizontal and vertical flipping and rotations (10, 30, 45 degrees), to enhance segmentation efficacy. The performance of the segmentation models was assessed on Set B of Silicodata, using metrics such as Intersection over Union (IOU) and mean IOU. For training, we maintained a batch size of 4 and optimized using Dice Loss for binary segmentation, conducting the training over 50 epochs on NVIDIA V100-DGXS GPUs.

### Classification Task

For the classification task, we have utilized six diverse deep Convolutional Neural Network (CNN) architectures - ResNet18^25^, InceptionV3^27^, VGG11^28^, AlexNet^29^, SqueezeNet^30^, and DenseNet121^31^ - all initialized with ImageNet pre-trained weights, alongside the Vision Transformer, ViT (base) with a 16 × 16 patch size, also pre-trained on the ImageNet dataset. These models were then fine-tuned on the complete Silicodata dataset. The Silicodata was partitioned into training, validation, and test sets with proportions of 70%, 10%, and 20%, respectively, and the classification tasks were conducted under two scenarios: a four-class multi-class setting (Silicosis, STB, TB, and normal) and a binary class setting (unhealthy and normal), with silicosis, STB, and TB samples combined under the ‘unhealthy’ label. Training was carried out over 50 epochs with a batch size of 64, using Cross-entropy loss and the Adam optimizer with a learning rate of 1e-4. The evaluation of model performance was facilitated through 5-fold cross-validation, with model selection based on the best validation set performance.

### Evaluation Results and Analysis

For assessing the performance of the segmentation models, Set B served as the test set. On the other hand, the evaluation of the classification task was conducted using a predefined test set that encompasses samples from both Set A and Set B of the complete dataset.

#### Segmentation Task

**Fig. 7:**
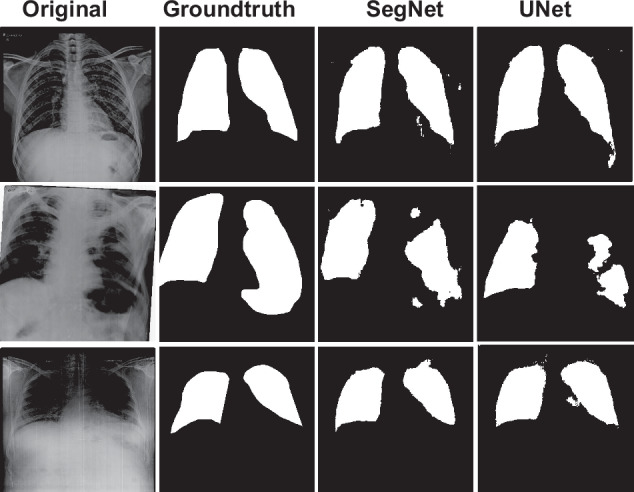
The results for prediction masks for both SegNet^23^, and U-Net^24^ Architecture. The above results showcase the challenges in segmenting the lung field from Silicosis and related disorder Silicotuberculosis. Samples of the Normal (Top row), silicotuberculosis (middle row) and Silicosis CXR (bottom row) show no proper demarcation in the affected cases. Undetected Silicosis worsens the lung tissue over the time and results in Silicotuberculosis.

The objective of the segmentation experiment is to assess how well current lung segmentation algorithms perform in identifying the lungs and regions of interest, particularly in cases of disease localization. Segmentation is crucial for precisely localizing diseases within the lung. However, the presence of diseases such as Silicosis and silicotuberculosis complicates this process due to the damage they cause to lung tissue, often obscuring the clear delineation of lung fields. Our findings indicate that the quality of the sample has a direct impact on segmentation accuracy, with normal lung samples being more straightforward to segment than those affected by Silicosis or silicotuberculosis. In illustrative results, the top row of Fig. 7 displays a normal input image alongside its ground truth mask and the predicted masks by two prominent deep learning models, UNet and SegNet. The predicted masks align more closely with the ground truth for normal samples compared to those with Silicotuberculosis, which presents significant challenges due to extensive lung tissue degradation. These segmentation outcomes highlight the importance of early Silicosis detection to mitigate the risk of developing further complications like TB or silicotuberculosis. The segmentation performance, measured by Intersection over Union (IOU) and mean IOU for Silicodata using both SegNet and UNet, is documented, revealing an approximate IOU of 0.86 for both models, also highlighted in Table 3. Visual comparisons in the figures suggest that UNet delivers superior segmentation results.

**Table 3:** Illustrates the Segmentation result for lung field with UNet^24^ and SegNet^23^.

Model	SegNet		U-Net	
**Classes**	Lung Region	Other Region	Lung Region	Other Region
**Mean IOU**	0.856		0.854	
**Class wise IOU**	0.794	0.917	0.792	0.915

#### Classification Task

The research protocol, as detailed in Table 2, has guided the execution of four-class classification experiments. The finetuned models’ efficacy is quantified through metrics such as the F1-score, Area Under the Curve (AUC), class-wise accuracy, and sensitivity at given specificities for two distinct thresholds, with a summary of these outcomes presented in Table 4. Notably, VGG11 emerged as a standout, delivering superior performance despite its simpler architecture. The ViT model, leveraging Transformer technology, excelled beyond traditional CNN-based models, except VGG11, by effectively capturing long-range dependencies, thus enhancing classification results in the context of limited sample sizes. However, across baseline approaches, a common shortfall is observed in disease detection accuracy when examining class-wise performance.

**Table 4:** Summarizing the average results obtained on the five-fold cross-validation on different existing deep learning models.

Model	Classes	F1-Score	Class-wise Accuracy	Class-wise AUC
VGG11^28^	Normal	**0.69**	0.75 ± 0.010	0.91 ± 0.001
	Tuberculosis		0.46 ± 0.090	0.75 ± 0.008
	Silicosis		0.54 ± 0.010	0.85 ± 0.005
	SilicoTuberculosis		0.67 ± 0.010	0.91 ± 0.006
	Normal		0.69 ± 0.004	0.89 ± 0.005
	Tuberculosis		0.48 ± 0.014	0.70 ± 0.008
	Silicosis		0.48 ± 0.015	0.82 ± 0.003
ResNet18^25^	SilicoTuberculosis	0.58	0.64 ± 0.007	0.87 ± 0.005
	Normal		0.76 ± 0.003	0.90 ± 0.003
	Tuberculosis		0.47 ± 0.160	0.76 ± 0.018
	Silicosis		0.52 ± 0.002	0.83 ± 0.005
AlexNet^29^	SilicoTuberculosis	0.64	0.59 ± 0.177	0.88 ± 0.004
	Normal		0.75 ± 0.010	0.91 ± 0.002
	Tuberculosis		0.50 ± 0.025	0.72 ± 0.015
	Silicosis		0.48 ± 0.018	0.80 ± 0.011
SqueezNet^30^	SilicoTuberculosis	0.58	0.48 ± 0.016	0.87 ± 0.004
	Normal		0.70 ± 0.020	0.89 ± 0.006
	Tuberculosis		0.52 ± 0.010	0.71 ± 0.005
	Silicosis		0.46 ± 0.020	0.77 ± 0.007
DenseNet121^31^	SilicoTuberculosis	0.54	0.43 ± 0.020	0.84 ± 0.013
	Normal		0.74 ± 0.009	0.87 ± 0.007
	Tuberculosis		0.45 ± 0.010	0.71 ± 0.004
	Silicosis		0.53 ± 0.007	0.81 ± 0.007
InceptionV3^27^	SilicoTuberculosis	0.60	0.59 ± 0.017	0.88 ± 0.006
	Normal		0.80 ± 0.011	0.93 ± 0.002
	Tuberculosis		0.57 ± 0.018	0.80 ± 0.013
	Silicosis		0.59 ± 0.031	0.87 ± 0.012
ViT^37^	SilicoTuberculosis	0.67	0.60 ± 0.031	0.90 ± 0.006

The bold values represent the best results achieved.

For binary classification, we adopted the same metrics as those used in the four-class scenario. According to Table 5, all seven baseline models demonstrated commendable F1-scores, exceeding 0.80. VGG11 distinguished itself across all evaluation criteria, achieving an AUC of 0.93 with minimal variance over five-fold cross-validation, marking it as the top performer among the baseline models. The analysis indicates consistent performance across models for Silicosis and STB detection, with normal CXRs generally identified with high accuracy, likely attributable to the high-quality nature of these samples. The diseased classes presented a challenge, with sample noise and co-existing findings complicating detection. Despite these challenges, ViT showcased robust performance across categories, with VGG11 and ResNet18 notably effective in identifying STB, even in the face of limited training data. This suggests that STB samples may possess more distinct features compared to TB and Silicosis, highlighting the potential for misdiagnosis between Silicosis and TB. Although all models reported solid AUC values, a balanced performance across classes remained elusive, with larger models like DenseNet121 and InceptionV3 underperforming due to potential overfitting issues arising from the small sample size. Sensitivity results, detailed in Table 6 for 90% and 99% specificity thresholds, corroborate these findings, with performance notably dropping at the 99% threshold.

**Table 5:** Performance for 2 class classification task.

Model	Classes	F1-Score	Class-wise Accuracy	Class-wise AUC
VGG11^28^	Normal	**0.864**	0.84 ± 0.005	0.93 ± 0.001
	unhealthy		0.87 ± 0.006	0.93 ± 0.001
ResNet18^25^	Normal	0.854	0.82 ± 0.010	0.92 ± 0.001
	unhealthy		0.87 ± 0.004	0.92 ± 0.001
AlexNet^29^	Normal	0.823	0.78 ± 0.011	0.92 ± 0.002
	unhealthy		0.84 ± 0.006	0.92 ± 0.002
SqueezeNet^30^	Normal	0.844	0.80 ± 0.008	0.92 ± 0.005
	unhealthy		0.87 ± 0.009	0.92 ± 0.005
DenseNet121^31^	Normal	0.856	0.82 ± 0.008	0.91 ± 0.003
	unhealthy		0.87 ± 0.005	0.91 ± 0.003
InceptionV3^27^	Normal	0.816	0.79 ± 0.010	0.89 ± 0.001
	unhealthy		0.83 ± 0.004	0.89 ± 0.002
ViT^37^	Normal	0.83	0.77 ± 0.004	0.90 ± 0.001
	unhealthy		0.87 ± 0.005	0.90 ± 0.001

The bold values represent the best results achieved.

**Table 6:** Illustrates the evaluation of existing deep learning algorithm for silicosis detection using proposed *Silicodata*.

Sensitivity @ Y Specificity	Classes	VGG11	DenseNet121	ResNet18	SqueezeNet	AlexNet	Inception V3	ViT
Y = 90%	Normal	0.74 ± 0.010	0.71 ± 0.160	0.68 ± 0.014	0.75 ± 0.007	0.71 ± 0.006	0.65 ± 0.006	0.82 ± 0.011
	TB	0.33 ± 0.180	0.30 ± 0.150	0.37 ± 0.015	0.34 ± 0.040	0.36 ± 0.012	0.32 ± 0.020	0.49 ± 0.027
	Silicosis	0.56 ± 0.030	0.37 ± 0.020	0.47 ± 0.015	0.45 ± 0.020	0.53 ± 0.019	0.52 ± 0.012	0.59 ± 0.028
	STB	0.74 ± 0.140	0.59 ± 0.034	0.60 ± 0.012	0.55 ± 0.015	0.57 ± 0.020	0.66 ± 0.023	0.67 ± 0.028
Y = 99%	Normal	0.44 ± 0.020	0.34 ± 0.022	0.30 ± 0.025	0.37 ± 0.033	0.48 ± 0.020	0.30 ± 0.031	0.40 ± 0.075
	TB	0.12 ± 0.019	0.07 ± 0.020	0.09 ± 0.012	0.07 ± 0.006	0.10 ± 0.010	0.08 ± 0.006	0.17 ± 0.019
	Silicoisis	0.24 ± 0.004	0.14 ± 0.018	0.16 ± 0.010	0.14 ± 0.017	0.12 ± 0.020	0.26 ± 0.019	0.29 ± 0.054
	STB	0.24 ± 0.023	0.17 ± 0.026	0.24 ± 0.022	0.09 ± 0.019	0.25 ± 0.021	0.16 ± 0.014	0.34 ± 0.052

To obtain sensitivity for each class, a one-vs-all strategy is followed.

In our experiments, we downscaled the images to a resolution of 224  × 224 pixels for classification and segmentation tasks. This was specifically done to enable fine-tuning on pre-trained models, which often require input images of standardized sizes due to architectural constraints. The reduced resolution allows for computational efficiency and compatibility with these models. However, we acknowledge that downscaling can lead to the loss or diminished visibility of subtle radiographic features, such as small nodules or opacities. During our experiments, we observed that reducing the image size sometimes resulted in these critical features being less prominent or even disappearing. This may have affected the performance of the models in detecting Silicosis-related abnormalities.

**Fig. 8:**
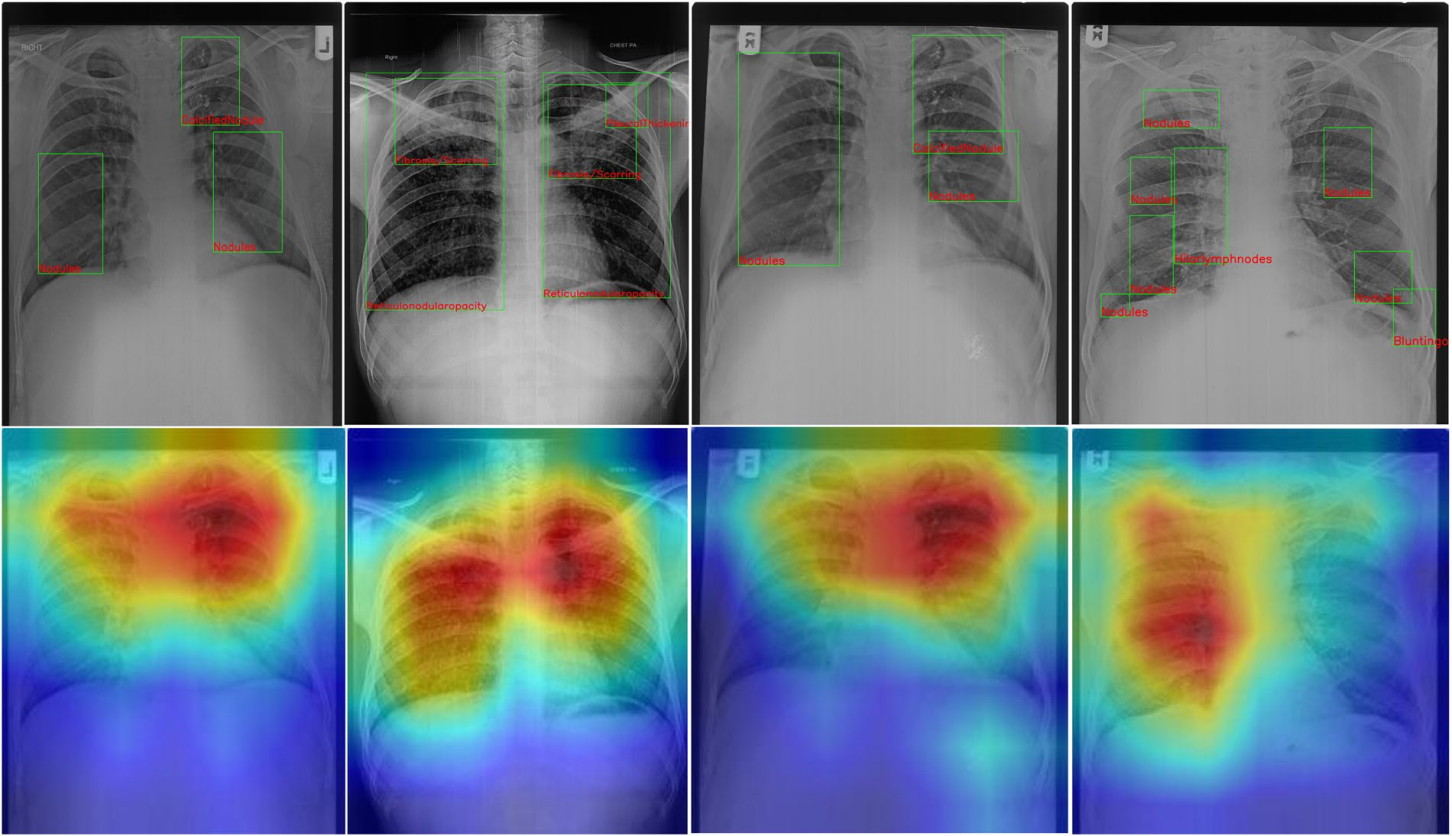
Showcasing the samples for CAM analysis using GradCAM^32^ for VGG11 model. The **top** row is the ground truth annotation depicting the local labels (manifestations) in the chest X-rays generated by the team of expert radiologists. The **bottom** row showcases the GradCAM outputs for each corresponding sample in the top row.

We also explored model explainability using GradCAM^32^, which shed light on the decision-making processes of models, particularly VGG11, as evidenced in Fig. 8. This not only emphasizes model transparency but also aligned closely with ground truth annotations, suggesting a significant potential for advancing Silicosis research within the constraints of limited data. For future, we aim to expand Silicodata with more samples and detailed expert annotations, fostering broader research into Silicosis and silicotuberculosis detection. Current studies on pneumoconiosis, especially Silicosis, have been limited to binary classifications on private datasets of small scale. We envision that Silicodata will catalyze further investigations, including report generation and advanced classification challenges, thereby contributing to the early diagnosis and understanding of Silicosis.

## Usage Notes

The dataset is hosted at Harvard Dataverse^19^. Requestors are required to provide a signed license agreement to gain access. The signed agreement, duly authorized by an official at the requestor’s organization, should be emailed to **databases@iab-rubric.org**. Access credentials will typically be provided within one week of submission. Researchers who use this dataset in publications must cite the original article.

## Data Availability

The segmentation models are implemented in the Pytorch framework. The pre-trained models are downloaded from the official website of Pytorch^33^. We used Python as a programming language and Pytorch as the deep-learning framework for experiments. The pretrained ViT is downloaded from (https://huggingface.co/docs/transformers/index) To load and resize the images, we used the OpenCV library^34^, and to report the results, we used the Scikit-learn library^35^. To plot the images, we used the Matplotlib library^36^. To reproduce the results, the implementation codes can be found at (https://github.com/IAB-IITJ/Silicodata).
